# Seizure Associated Takotsubo Syndrome: A Rare Combination

**DOI:** 10.1155/2017/8458054

**Published:** 2017-07-24

**Authors:** Htay Htay Kyi, Nour Aljariri Alhesan, Sunil Upadhaya, Samer Al Hadidi

**Affiliations:** ^1^Hurley Medical Center, Flint, MI, USA; ^2^Hurley Medical Center/Michigan State University, Flint, MI, USA

## Abstract

Takotsubo cardiomyopathy (TC) is increasingly recognized in neurocritical care population especially in postmenopausal females. We are presenting a 61-year-old African American female with past medical history of epilepsy, bipolar disorder, and hypertension who presented with multiple episodes of seizures due to noncompliance with antiepileptic medications. She was on telemetry which showed ST alarm. Electrocardiogram (ECG) was ordered and showed ST elevation in anterolateral leads and troponins were positive. Subsequently Takotsubo cardiomyopathy was diagnosed by left ventriculography findings and absence of angiographic evidence of obstructive coronary artery disease. Echocardiogram showed apical hypokinesia, ejection fraction of 40%, and systolic anterior motion of mitral valve with hyperdynamic left ventricle, in the absence of intracoronary thrombus formation in the angiogram. Electroencephalography showed evidence of generalized tonic-clonic seizure. She was treated with supportive therapy. This case illustrates importance of ECG in all patients with seizure irrespective of cardiac symptoms as TC could be the cause of Sudden Unexpected Death in Epilepsy (SUDEP) and may be underdiagnosed and so undertreated.

## 1. Background 

Takotsubo cardiomyopathy (TC) is increasingly recognized in neurocritical care population especially in postmenopausal females, with subarachnoid haemorrhage being the most common trigger in neurocritical care unit and seizure being the second common cause [1]. This association is underreported and could possibly be a cause of Sudden Unexpected Death in Epilepsy (SUDEP), but if early diagnosed and properly treated, it is associated with low mortality and is considered reversible in most of the cases.

## 2. Case Presentation 

**Figure 1:**
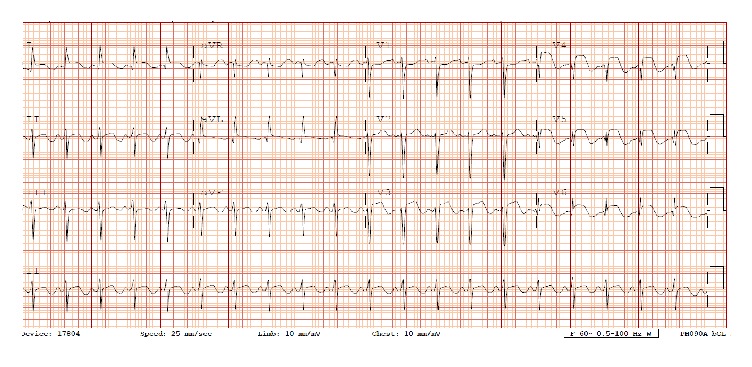
ECG.

**Figure 2:**
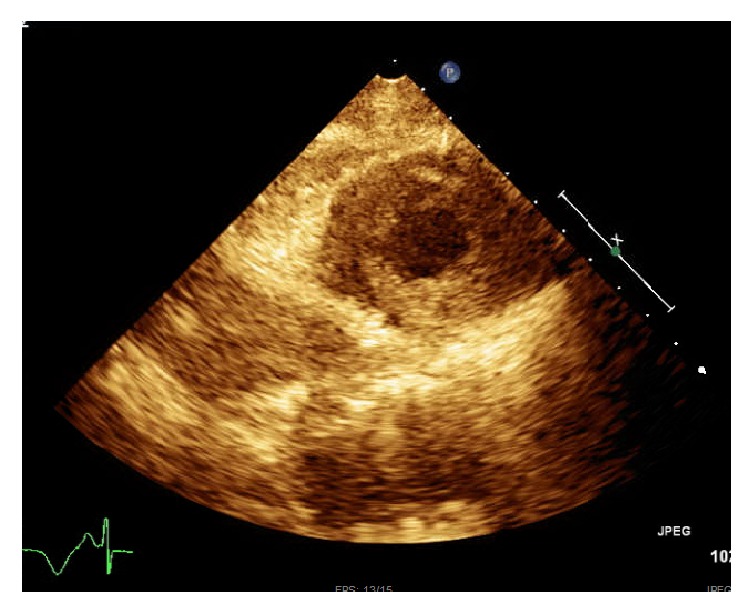
Echocardiogram showing apical hypokinesia.

**Figure 3:**
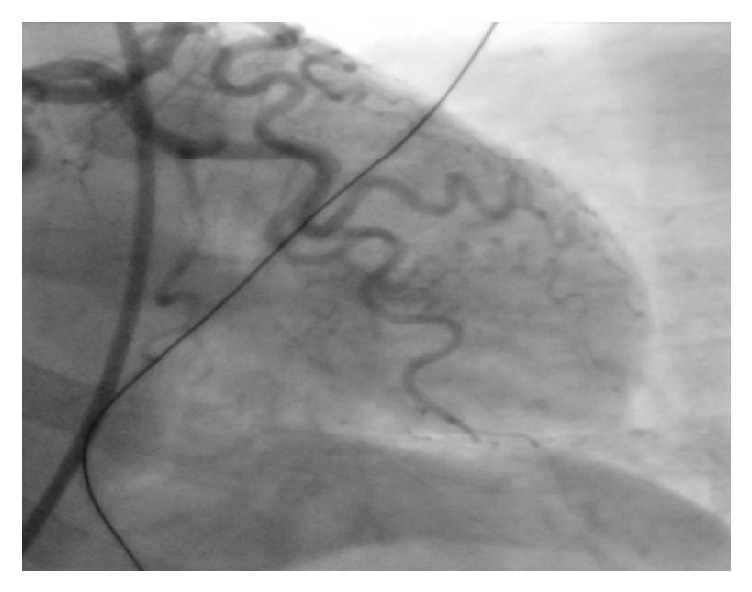
Coronary angiogram and left ventriculography.

A 61-year-old African American lady with history of epilepsy, bipolar disorder, and hypertension admitted with recurrent tonic-clonic seizures secondary to medication noncompliance. She denied chest pain, shortness of breath, and palpitations. She had no family history of premature coronary artery disease (CAD) or cardiomyopathy. She is a former smoker. She denied alcohol and drug abuse. She was placed under telemetry upon admission and ST elevation alarm was activated after few hours of admission. Her ECG showed ST elevation in anterolateral leads (Figure 1) and troponins I peak to 38.97 ng/mL. Echocardiogram (Figure 2) showed apical hypokinesia, ejection fraction of 40%, and systolic anterior motion of mitral valve with hyperdynamic left ventricle, in the absence of intracoronary thrombus formation in the angiogram (Figure 3). Subsequently patient underwent coronary angiography which showed no evidence of CAD, hypokinetic apex, and ejection fraction of 55%, which were consistent with TC. Electroencephalogram (EEG) showed evidence of generalized tonic-clonic seizure. Magnetic resonance imaging (MRI) demonstrated areas of restricted diffusion in left hippocampal gyrus, right side of splenium of corpus callosum, and bilateral temporal lobes related to seizures/postictal status. She was treated with supportive therapy, aspirin, and beta-blocker, angiotensin converting enzyme inhibitor, statin, and Depakote. She stabilized without complications. Her seizure was controlled with Depakote.

## 3. Discussion

The term “Takotsubo” was first described in 1990 in Japan, taken from the Japanese name for an octopus' trap, which has a shape that is similar to the systolic apical ballooning appearance of the LV. It is also called stress cardiomyopathy, apical ballooning syndrome, or broken heart syndrome [12]. Since then, many cases of seizure related TC have been reported [2, 13–26]. As per the data of International Takotsubo Registry which included 1750 patients with stress cardiomyopathy, 89.8 percent were women and mean age was 66.4 years [27]. About 27.7% of the patients had emotional triggers compared to 36% incidence of physical triggers. In addition, about 28.5% of patient did not have any evidence of trigger. In one series of 3265 patients with troponin positive acute coronary syndrome, about 1% of patient actually had TC [28]. Diagnosis of Takotsubo cardiomyopathy is based on seven diagnostic criteria which include anatomical features, ECG changes, cardiac biomarkers, and reversibility of the myocardial dysfunction [29].

In a case series of 15 patients, about 33.3% were associated with seizures, all of them being generalized type [30]. In a study, there were about 39 cases of seizure related TC patients compared to patients with TSS which was related to causes other than seizure, and it was found that seizure related TC patients were younger (61.5 versus 68.5 years, *p* < 0.0001), were more frequently males (17 versus 9%, *p* value of 0.004), and had less frequently chest pain (6 versus 76%, *p* value of <0.005), higher risk of cardiogenic shock (25 versus 8%, *p* value of 0.003), and higher risk of recurrence (14 versus 3%, *p* value of 0.004) [8]. Few studies have shown increment in catecholamine associated with seizure activities [31, 32].

In a review which evaluated the possibility of seizure related TC as a cause of SUDEP, 74 cases were described, age was reported to be between 18 and 82 years, 86% were females, and 14% were males; the outcome was reported in 63 of the 74, with full recovery in 97% of the cases, incomplete recovery in none of the patients, and fatal outcome in 3% of cases [33]. The authors concluded no relation exists between TC and SUDEP.

It was also found that cardiac arrhythmia and conduction abnormalities were more common in seizure related TC compared to other triggers; the severe clinical picture of seizure related TC is probably related to excess catecholamine surge in those with intractable epilepsy [8]. It is also worth mentioning that patients with seizure related TC rarely present with typical chest pain which could be related to impaired consciousness or sedation due to antiepileptic medications, and given the fact that Sudden Unexpected Death in Epilepsy accounts for 5–30% of all deaths in epilepsy patients, ECG or echocardiogram would be of great help in ruling out TC and early management of TC or related fatal arrhythmia [8, 33].

Our patient had both typical and atypical features of TC as she was a postmenopausal female who presented with multiple episodes of seizures with history of underlying epilepsy. Her ECG showed ST-segment elevation and echocardiogram showed ejection fraction of 40%. Patients with typical TC more often presented with an acute psychiatric episode than those with atypical TC (10.5% versus 6.3%) [34], whereas patients with an atypical form presented more often with a neurologic disorder (29.6% versus 22.9%) [34]. Atypical TC accounts for nearly 20% of all cases of TC and has different clinical features than typical TC, including younger patient age, higher frequency of ST-segment depression, higher prevalence of neurologic disease, and less impaired left ventricle ejection fraction [34].

Our case was similar to previously reported cases of seizure related TC (Table 1), in the fact that our patient was a postmenopausal female who had incidental finding of ST elevation on telemetry and positive troponins with no chest pain or any cardiac symptoms. The setting was that of an uncontrolled generalized tonic-clonic seizure with characteristic echocardiographic and coronary angiography features and the outcome was favorable and she fully recovered with no complications.

This case illustrates importance of ECG in all patients with seizure irrespective of cardiac symptoms as TC could be the cause of SUDEP and may be underdiagnosed and so undertreated. Diagnosing TC in patients after a seizure is essential since treatment of TC may improve the outcome of affected patients. In general, postmenopausal women who present with seizure are admitted to neurocritical care unit for other reasons including strokes and subarachnoid haemorrhage, are at increased risk of TC, and should have baseline ECG ordered and repeat ECG ordered in cases of change in clinical condition, and also we should keep in mind that in this particular group the presentation may be atypical since patients are under the effect of sedatives and antiepileptic medications.

## Figures and Tables

**Table 1:** Cases of seizure related TC.

Case report	Sex	Age	History of heart disease	Chest pain	Seizure	*ECG*	* Troponin peak (ng/ml)*	Echo	Coronary angiography	Complications during hospitalization	Clinical outcome
Kalra et al. [2]	F	25	No	No	Generalized	*Inverted T wave in II,III, AVF, and V3–V6, long QT interval*	* N.A.*	Hypokinesia of left ventricle apex	Normal	None	Favorable
Johar et al. [3]	F	57	No	Yes	No	*ST elevation in V1–V4*	* 17.11*	Marked akinesis of mid-to-distal septum, apex and anterior wall, EF 30%	Normal coronary arteries without any stenosis or obstruction	None	Favorable
Lemke et al. [1]	F	50	No	No	Generalized	*ST depression in V2-V3*	* 1*	Apical wall hypokinesis, EF 35–40%	Normal	Hypotension, acute pulmonary edema	Favorable, discharged home on hospital day 5
Chin et al. [4]	M	62	No	No	Generalized	*ST depression in V2–V4*	* 17.8*	Anterior, lateral, distal inferior wall hypokinesis. Normal apical mobility, EF 40%	Normal	Hypotension, acute pulmonary edema	Favorable
Chin et al. [4]	F	50	No	No	Generalized	*Inverted T wave in V1–V4*	* 3*	Mid left ventricular hypokinesis, hyperdynamic function of apex and basis	Normal	None	Favorable
Bosca et al. [5]	F	61	No	Yes	Generalized	*ST elevation in V3, inverted T waves in V4–V6 *	* Mild elevation*	Lateral and apical wall dyskinesis with LVEF impairment	Normal	Acute heart failure	Favorable
Sakuragi et al. [6]	F	59	Sick sinus syndrome. PM	No	Generalized, status epilepticus	*ST elevation in I, aVL, V2–V4, long QT interval*	* N.A. (CPK mb: 39 UI/L)*	Severe anterior and apical wall hypokinesis. Mild LVEF impairment	Normal	Heart failure	Favorable
Shimizu et al. [7]	F	75	No	No	Generalized	*ST elevation in II, III, aVF, V2–V5 *	* Positive qualitative test*	Apical ballooning, basal hyperkinesis	Normal left coronary, right coronary hypoplasia, positive spasm test	Hypotension	Favorable, discharged on hospitalization day 27
Stöllberger et al. [8]	F	71	No	No	Generalized	*Q waves and ST elevation in II, III, aVF, V5-V6 *	* Positive*	Apical, anteroapical and inferoapical akinesis	Normal	Left ventricular rupture	Death on hospitalization day 3
Yousuf et al. [9]	F	58	No	No	Generalized	*ST elevation in V2–V6*	* 10*	Anterior, septal, apical akinesis, ballooning	Normal	Acute pulmonary edema, kidney injury, rhabdomyolysis	Complete recovery in 1 week
Wakabayashi et al. [10]	F	68	No	No	Generalized, status epilepticus	*Inverted T waves in I, II, III, aVF, V2–V6, long QT interval*	* N.A.*	Apical ballooning with hypokinesis	—	Left ventricle apical thrombus	Favorable, discharged on hospital day 44
Seow et al. [11]	M	62	No	No	Generalized, status epilepticus	*Anterior derivations ST elevation*	* Elevation*	Mild ventricular ballooning, sparing of the apex, EF 40%	Nonsignificant stenosis in the obtuse marginal branch of Cx	Hypotension	Favorable
